# Respiratory Health before and after the Opening of a Road Traffic Tunnel: A Planned Evaluation

**DOI:** 10.1371/journal.pone.0048921

**Published:** 2012-11-29

**Authors:** Christine T. Cowie, Nectarios Rose, Wafaa Ezz, Wei Xuan, Adriana Cortes-Waterman, Elena Belousova, Brett G. Toelle, Vicky Sheppeard, Guy B. Marks

**Affiliations:** 1 Woolcock Institute of Medical Research, Sydney, Australia; 2 Cooperative Research Centre for Asthma and Airways, Sydney, Australia; 3 NSW Health Department, Sydney, Australia; 4 Western Sydney and Nepean Blue Mountains Public Health Unit, Sydney, Australia; Louisiana State University Health Sciences Center, United States of America

## Abstract

**Objective:**

The construction of a new road tunnel in Sydney, Australia, and concomitant reduction in traffic on a major road presented the opportunity to study the effects of this traffic intervention on respiratory health.

**Methods:**

We made measurements in a cohort of residents in the year before the tunnel opened (2006) and in each of two years afterwards (2007–2008). Cohort members resided in one of four exposure zones, including a control zone. Each year, a respiratory questionnaire was administered (n = 2,978) and a panel sub-cohort (n = 380) performed spirometry once and recorded peak expiratory flow and symptoms twice daily for nine weeks.

**Results:**

There was no consistent evidence of improvement in respiratory health in residents living along the bypassed main road, despite a reduction in traffic from 90,000 to 45,000 vpd. Residents living near tunnel feeder roads reported more upper respiratory symptoms in the survey but not in the panel sub-cohort. Residents living around the tunnel ventilation stack reported more upper and lower respiratory symptoms and had lower spirometric volumes after the tunnel opened. Air pollutant levels measured near the stack did not increase over the study period.

**Conclusion:**

The finding of adverse health effects among residents living around the stack is unexpected and difficult to explain, but might be due to unmeasured pollutants or risk factors or an unrecognized pollutant source nearby. The lack of improvement in respiratory health among people living along the bypassed main road probably reflects a minimal change in exposure due to distance of residence from the road.

## Introduction

Road tunnels are increasingly being constructed in major world cities to help alleviate traffic congestion. They are often located within densely populated areas, have high traffic volumes, including a substantial proportion of heavy duty vehicles, and pollutants are commonly vented to the external environment through stacks [1]–[3]. However, studies investigating the health impact of traffic-related air pollution (TRAP) on communities residing around road tunnels, or their ventilation stacks and portals, are scarce, probably due to difficulty in disentangling health effects associated with general traffic from effects attributable to emissions arising in the tunnels [4].

We are aware of only one study that has investigated health effects associated with exposure to road tunnel stack emissions [2]. This cross-sectional study, conducted after the tunnel was opened, found no difference in the prevalence of self-reported respiratory and irritant symptoms between three zones estimated to have varying exposure to the ventilation stack plume.

The Lane Cove Tunnel (LCT) is a 3.6 km long road tunnel that connects two motorways and is vented by two stacks located near each end of the tunnel. Modelling prior to tunnel construction predicted that areas around the tunnel feeder roads would experience increased exposure to nitrogen dioxide (NO_2_) and particulate matter (PM), and areas adjacent to the bypassed main road would experience a decrease in exposure [5]. Modelling of stack emissions forecast little detectable impact on the surrounding area. The tunnel commenced operation on 25 March 2007, with an average of 43,446 vehicles per day (vpd) during May, 2007 increasing to 58,218 vpd by November, 2008, with 2.9% and 3.5% of vehicles respectively classified as heavy or articulated vehicles. Opening of the tunnel was accompanied by an approximate halving of vehicle numbers from 90,000 vpd along the bypassed main road [6].

**Figure 1:**
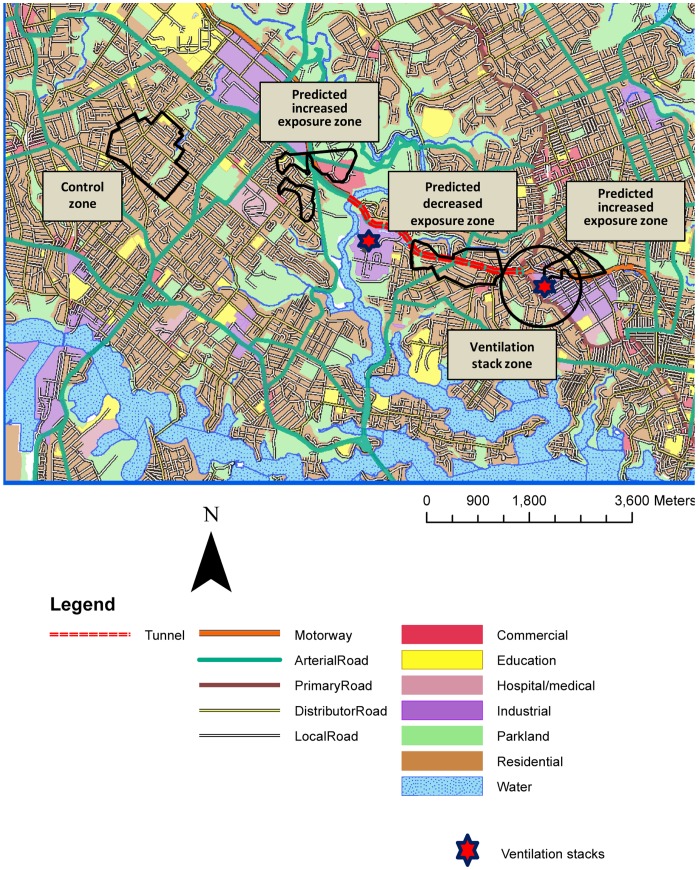
Map of study area showing the zones, tunnel, ventilation stacks and land use.

This study was commissioned by the NSW Health Department to inform public policy on road tunnels and their ventilation stacks and to opportunistically study respiratory health effects of a substantial traffic intervention. The principal hypothesis was that, after tunnel opening, adverse respiratory health outcomes would increase among residents near the tunnel feeder roads and decrease among residents along the bypassed main road, after adjustment for concurrent changes in a control zone. A subsidiary hypothesis was that people living around the ventilation stack would not experience a change in respiratory health status.

**Figure 2:**
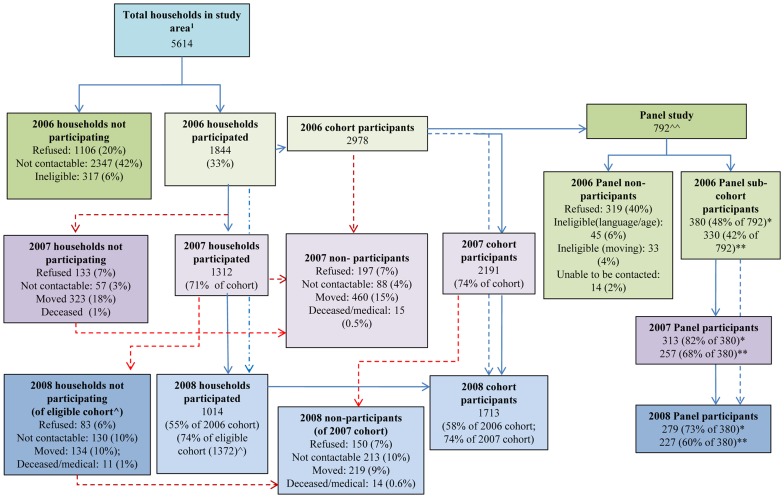
Participation flowchart. ^1^ Estimate derived from census of households within the study area. ^∧^ Eligible cohort = 2007 cohort + additional households potentially eligible to be interviewed in 2008. ^∧∧^ Subjects approached to participate in panel study. ^*^ Subjects who took part in spirometry and eNO measurement. ^**^ Subjects who took part in daily lung function and symptom diary. Blue arrow: Household and participant participation. Red arrow: Loss to follow-up.

## Methods

### Ethics Statement

Approval for the study was obtained from the University of Sydney Human Research Ethics Committee (HREC). Recruitment of subjects occurred through a household doorknock survey. At the time of the home visit an introductory letter about the study was provided to an adult resident, the nature of the study was discussed, and consent to participate in the survey was sought. If consent was given, this adult was considered to be the “primary respondent” for the household, and was interviewed using a standardised questionnaire. Parents completed questionnaires for children less than 16 years, and children aged 16–18 years completed questionnaires with a parent/caregiver present. Self-completion questionnaires were left for other household adults not present at the time of the visit. Up to three follow-up phone calls were made for return of the self-completed questionnaires. Interviewers returned to households up to three times and left calling cards for households unable to be contacted. The household contact and interviewing process was documented using a standardised form. Participants in the panel sub-cohort provided separate and additional written consent. The recruitment process for the whole cohort and sub-cohort was approved by the HREC.

**Table 1:** Symptom prevalence and other characteristics of the study cohort by zone and year (n = 2978).

	Reduced exposure zone			Increased exposure zone^a^			Eastern stack zone^a^			Control zone		
	2006	2007	2008	2006	2007	2008	2006	2007	2008	2006	2007	2008
**n**	1024	756	608	589	422	302	645	500	394	768	537	424
**(% of 2006)**		(74)	(59)		(72)	(51)		(78)	(61)		(70)	(55)
**%**												
Adult (age ≥18 yrs)	72	70	68	74	72	71	75	72	72	72	72	72
Female	56	57	58	53	54	57	54	53	54	53	54	54
Diagnosed asthma	18	20	20	18	24	21	16	19	21	15	17	19
Current asthma	11	13	13	12	14	12	9	12	12	9	10	9
Wheeze	15	18	15	14	19	18	11	20	17	10	13	13
Asthma medication	11	14	15	11	14	14	10	14	13	9	10	11
Inhaled corticosteroids	6	8	9	4	8	7	4	7	6	5	5	7
Cough	40	49	50	33	52	48	36	52	53	34	47	48
Lower resp. symptoms^b^	39	47	47	35	51	50	34	48	52	33	43	43
Severe lower resp. symptoms^b^	10	11	12	12	14	13	8	17	12	8	9	10
Upper resp. symptoms^b^	17	24	22	10	25	28	12	27	25	12	22	20
Mouth symptoms	14	17	18	9	21	21	11	24	22	13	17	18
Smoker c	7	6	6	12	10	9	11	9	7	12	13	10
***Home environmental factors***												
Unflued gas heater	25	28	33	12	21	25	18	25	27	20	24	29
Gas cooktop or oven	52	58	59	25	52	51	44	56	56	42	46	48
ETS^d^ at home	7	3	4	14	9	10	8	5	5	8	5	7
***Educational status***												
Tertiary educated	61	65	64	46	46	44	62	61	61	44	42	36
High school/diploma^e^	31	28	28	43	38	41	31	34	31	43	38	46
Up to middle school^f^	8	8	8	11	16	15	7	6	7	13	19	18
***Work status***												
Paid Work	74	76	77	66	70	70	75	76	78	71	70	73

a Participants in overlapping area of increased exposure zone and stack zone, entire cohort (2006 = 48; 2007 = 24; 2008 = 15) contributed data to both zones.

b Composite variables derived from questionnaire data (see Supporting information, Table S1).

c Subjects <18 years assumed to be non-smokers.

d Environmental tobacco smoke.

e Included Diploma/TAFE, and participants who responded “Other”.

f Included participants who refused to respond.

### Subject Recruitment and Exposure Assignment

We recruited a cohort of 2,978 participants during June-December 2006 (pre-tunnel opening) and followed them up during the same time of year in 2007 and 2008 (post-tunnel opening), within two weeks of their initial participation date. Participants were recruited by door-knocking all households within four exposure zones (Figure 1), defined as follows:

- a zone along the bypassed main road (predicted ‘*decreased exposure zone’*);
- a zone around the tunnel feeder roads (predicted *‘increased exposure zone’*);
- a zone of 650 m radius around the tunnel’s eastern ventilation stack (*‘stack zone’*); and,
- a control zone.

**Table 2:** Lung function measures, and other characteristics of the diary panel sub-cohort, by zone and year (n = 380)^a^.

	Reduced exposure zone					Increased exposure zone^b^				Eastern stack zone^b^			Control zone		
	2006	2007		2008		2006		2007	2008	2006	2007	2008	2006	2007	2008
**n** c **(% of 2006)**	93	85 (91)		79 (85)		98		77 (78)	70 (71)	97	85 (88)	74 (76)	98	71(72)	61(62)
**%**															
Adult	46	42		41		48		44	43	45	42	46	52	54	56
Female	54	52		53		51		52	47	56	54	54	45	46	44
Atopic		58		60				57	53		50	48		58	58
**Mean (SD)**															
FEV_1_ pre-bd^d^ (L)	2.6 (0.8)	2.7 (0.8)		2.8 (0.8)		2.5 (0.9)		2.6(0.9)	2.8 (0.9)	2.8 (0.8)	2.9 (0.8)	3.1(0.8)	2.6 (0.9)	2.7 (0.8)	2.7 (0.7)
FEV_1_ post-bd (L)	2.7 (0.8)	2.8 (0.8)		2.9 (0.8)		2.6 (0.9)		2.7(0.9)	2.8 (0.9)	2.9 (0.9)	3.0 (0.8)	3.1(0.8)	2.7 (0.9)	2.8 (0.8)	2.8 (0.8)
FVC pre-bd (L)	3.2 (1.1)	3.4 (1.1)		3.5(1.1)		3.1 (1.2)		3.3(1.2)	3.4 (1.1)	3.3 (1.1)	3.6 (1.1)	3.8(1.1)	3.2 (1.1)	3.4 (1.0)	3.4 (0.9)
FVC post-bd (L)	3.3 (1.1)	3.4 (1.1)		3.5 (1.1)		3.1 (1.2)		3.3(1.2)	3.4 (1.1)	3.4 (1.2)	3.6 (1.1)	3.8(1.1)	3.2 (1.1)	3.4 (1.0)	3.4 (1.0)
eNO (ppb)^e^	9.7 (1.8)	17.8(2.0)		8.9 (2.3)		8.9 (1.9)		18.7(1.8)	9.7 (2.0)	9.2 (2.0)	16.3(1.7)	8.0(2.0)	8.9 (1.8)	17.9(1.8)	9.1 (1.8)
**n** f **(% of 2006)**	86	76 (88)		70 (81)		82		57(70)	52 (63)	85	69 (81)	62(73)	81	57 (70)	46 (57)
**Mean (SD)**															
PEF am (L/min)^g^	453 (106)		456 (94)		447 (98)		372 (78)	377 (72)	351 (83)	429(137)	420(131)	459(125)	382(110)	377(103)	379 (86)
PEF pm (L/min)^g^	450 (108)		462 (90)		453 (97)		377 (83)	386 (79)	355 (80)	420(136)	429(126)	460 (136)	396 (103)	378 (97)	379 (87)

a Each subject contributed up to 63 days of data in the diary panel.

b Participants in overlapping area of increased exposure zone and stack zone: diary panel cohort (2006 = 6(4); 2007 = 5(2); 2008 = 5(3)) contributed data to both zones.

c Participants with spirometry and eNO.

d bd-bronchodilator.

e Geometric mean and SD.

f Participants with PEF/symptom diary.

g PEF recorded three times twice daily at morning and evening. Highest of three measurements used for each session.

The first two zones were defined using NO_2_ contours from Cal3qhc and Calmet/Calpuff dispersion modelling (see Supporting Information, Appendix S1). The zone around the eastern stack was included to address community concern about stack emissions, and the radius of 650 m was chosen to ensure a sufficient sample size. The control zone was in a nearby suburb, away from the influence of the tunnel, and not expected to experience any change in TRAP.

**Table 3:** Symptom prevalence of the diary panel sub-cohort, by zone and year (n = 380)^a^.

	Reduced exposure zone			Increased exposure zone^b^			Eastern stack zone^b^			Control zone		
	2006	2007	2008	2006	2007	2008	2006	2007	2008	2006	2007	2008
**N** c (% of 2006)	86	76 (88)	70 (81)	82	57 (70)	52 (63)	85	69 (81)	62 (73)	81	57 (70)	46 (57)
**%**												
***Symptom days*** d												
At least once	99	96	94	96	93	98	95	97	94	94	91	98
10% of days	93	84	79	89	70	77	82	78	89	84	72	76
>20% of days	69	67	60	73	53	62	62	64	66	63	60	61
***Day-time symptoms*** d												
At least once	79	72	70	84	70	69	67	65	63	63	58	65
>10% of days	52	43	53	63	42	48	49	46	48	41	40	43
>20% of days	36	33	37	39	26	37	33	32	31	23	25	30
***Night-time symptoms*** d												
At least once	64	61	59	73	44	50	66	59	65	54	42	52
>10% of days	36	30	27	38	25	29	34	32	40	30	26	28
>20% of days	19	13	13	21	12	15	18	22	21	15	19	17
***Wheeze*** d												
At least once	28	24	20	35	21	23	22	28	35	35	28	33
>10% of days	9	7	9	17	4	10	11	9	5	15	14	7
>20% of days	3	7	3	10	4	4	6	4	0	11	9	4
***Bronchodilator use*** d												
At least once	12	16	10	21	11	12	6	7	8	12	12	13
>10% of days	3	7	6	12	7	10	2	3	2	6	11	4
>20% of days	2	1	1	5	5	4	0	0	0	5	9	4
***Cough*** d												
At least once	71	68	60	77	53	56	66	64	58	57	46	63
>10% of days	43	33	39	48	32	35	40	38	45	36	32	37
>20% of days	26	18	23	32	12	21	21	23	26	20	25	30
***Upper resp. symptoms*** d												
At least once	50	50	53	60	44	44	48	49	53	40	44	50
>10% of days	19	17	29	24	11	21	18	23	18	9	14	26
>20% of days	5	8	13	12	7	10	6	9	3	5	11	15
												
Lower resp symptomse f	37	44	33	26	57	51	38	32	44	31	38	39
Severe lower resp sympe f	6	7	8	12	16	13	5	7	6	9	7	12
Upper resp. symptoms e f	12	18	18	5	27	33	11	22	15	8	26	20
Mouth symptoms f	12	14	22	9	18	15	7	22	21	8	14	9

a Each subject contributed up to 63 days of data in the diary study.

b Participants in overlapping area of increased exposure zone and stack zone, diary panel cohort (2006 = 6(4); 2007 = 5(2); 2008 = 5(3)), contributed data to both zones. n =  subjects in overlapping area with spirometry; (n) = subjects in overlapping area taking part in peak flow and symptom diary.

c Panel sub-cohort participants who took part in peak flow/symptom diary.

d Symptoms recorded by diary. % represents proportion of subjects experiencing those symptoms a) at least one day during the study period; b) >10% of days during the study period; c) >20% of days during the study period.

e Composite variables derived from variables measured in questionnaire (See Table S1, Supporting Information).

f Attributes measured by questionnaire.

Eligibility criteria included: all residents within the zones; ages 2–75 years; being at home for ≥1 hour during rush hours (7∶00 am–9.30 am or 4.30 pm–7∶00 pm); and having sufficient English. Subjects were not told which zone they resided in. Participants were interviewed using the adult or children’s version of the questionnaire.

**Table 4:** Odds ratios for symptoms reported by questionnaire in 2007 and in 2008 relative to 2006 adjusted for change in the control zone.

Outcomes measured by questionnaire		2007 vs 2006		2008 vs 2006	
		Odds Ratio (95% CI)	P	Odds Ratio (95% CI)	P
***Upper respiratory symptoms***					
Reduced exp zone	Baseline model	0.7 (0.5, 1.2)	0.2	0.8 (0.5, 1.3)	0.3
	Adjusted	0.7 (0.4, 1.2)^a^	0.2	0.8 (0.5, 1.3)^a^	0.3
Increased exp zone	Baseline model	2.0 (1.2, 3.6)	0.02	3.2 (1.7, 6.1)	<0.001
	Adjusted	1.9 (1.0, 3.3)^a^	0.04	3.0 (1.6, 5.6)^a^	<0.001
Stack zone	Baseline model	1.8 (1.1, 3.2)	0.03	1.8 (1.0, 3.2)	0.05
	Adjusted	1.8 (1.1, 3.1)^a^	0.03	1.7 (1.0, 3.0)^a^	0.07
***Lower respiratory symptoms***					
Reduced exp zone	Baseline model	0.9 (0.6, 1.3)	0.7	0.9 (0.6, 1.4)	0.7
	Adjusted	0.9 (0.6, 1.3)^b^	0.6	0.9 (0.6, 1.4)^c^	0.6
Increased exp zone	Baseline model	1.5 (1.0, 2.2)	0.07	1.4 (0.8, 2.2)	0.2
	Adjusted	1.5 (1.0, 2.2)^b^	0.08	1.4 (0.8, 2.2)^c^	0.2
Stack zone	Baseline model	1.2 (0.8, 1.7)	0.5	1.6 (1.0, 2.4)	0.05
	Adjusted	1.2 (0.8, 1.7)^b^	0.4	1.6 (1.0, 2.5)^c^	0.04
***Severe lower respiratory symptoms***					
Reduced exp zone	Baseline model	1.1 (0.6, 2.0)	0.9	1.0 (0.5, 1.9)	1.0
	Adjusted	1.1 (0.6, 2.0)^d^	0.9	1.0 (0.5, 2.0)^d^	0.9
Increased exp zone	Baseline model	1.2 (0.6, 2.4)	0.7	1.0 (0.5, 2.1)	1.0
	Adjusted	1.1 (0.5, 2.2)^d^	0.8	1.0 (0.5, 2.0)^d^	0.9
Stack zone	Baseline model	3.0 (1.5, 6.0)	0.002	1.4 (0.7, 2.8)	0.4
	Adjusted	3.2 (1.6, 6.5)^d^	0.001	1.6 (0.8, 3.3)^d^	0.2
***Wheeze in last 3 months***					
Reduced exp zone	Baseline model	0.9 (0.5, 1.5)	0.7	0.9 (0.5, 1.5)	0.7
	Adjusted	0.9 (0.5, 1.6)^e^	0.8	0.7 (0.3, 1.2)^f^	0.2
Increased exp zone	Baseline model	1.1 (0.6, 2.0)	0.8	1.1 (0.6, 2.0)	0.8
	Adjusted	1.2 (0.6, 2.2)^e^	0.6	1.1 (0.5, 2.4)^f^	0.7
Stack zone	Baseline model	1.8 (1.0, 3.3)	0.06	1.8 (1.0, 3.3)	0.06
	Adjusted	2.0 (1.1, 3.6)^e^	0.03	1.5 (0.7, 3.0)^f^	0.3
***Cough in last 3 months***					
Reduced exp zone	Baseline model	0.8 (0.6, 1.1)	0.2	0.9 (0.6, 1.3)	0.4
	Adjusted	0.8 (0.6, 1.1)^g^	0.2	0.9 (0.6, 1.3)^h^	0.5
Increased exp zone	Baseline model	1.4 (0.9, 2.0)	0.1	1.1 (0.7, 1.8)	0.6
	Adjusted	1.5 (1.0, 2.2)^g^	0.06	1.2 (0.8, 1.9)^h^	0.4
Stack zone	Baseline model	1.1 (0.8, 1.7)	0.5	1.2 (0.8, 1.9)	0.3
	Adjusted	1.1 (0.8, 1.7)^g^	0.5	1.3 (0.8, 1.9)^h^	0.3

a Adjusted for: asthma, gas cooker or oven.

b Adjusted for: age, age^2^, gender, asthma, smoker, unflued gas heater.

c Adjusted for: age, age^2^, asthma, smoker, unflued gas heater.

d Adjusted for: age, age^2^, asthma, smoker, ETS at home.

e Adjusted for: asthma, unflued gas heater.

f Adjusted for: age, age^2^, asthma, unflued gas heater.

g Adjusted for: age, age^2^, asthma, smoker, gas cooker or oven.

h Adjusted for: age, age^2^, gender, asthma, smoker, gas cooker or oven.

A panel sub-cohort was recruited sequentially from the entire cohort to keep a nine week diary of symptoms and lung function. We originally intended recruiting only children to the sub-cohort, but, because of the scheduled opening of the tunnel and slower than expected recruitment, we decided, in late 2006, to also recruit adults. We recruited adults and children into two approximately equal strata until the sub-cohort quota was filled.

**Table 5:** Differences in spirometry and peak flow and odds ratios for eNO, in 2007 and 2008 relative to 2006, adjusted for change in the control zone (n = 380).

Lung function outcomes		2007 vs 2006		2008 vs 2006	
		Difference (95% CI)	P	Difference (95% CI)	P
***FEV1-pre bronchodilator (L)*** a b					
Reduced exp zone	Baseline model	−0.002 (−0.06, 0.06)	1.0	−0.01 (−0.08, 0.07)	0.9
	Adjusted	−0.002 (−0.06, 0.06)	1.0	−0.001 (−0.08, 0.08)	1.0
Increased exp zone	Baseline model	−0.02 (−0.08, 0.04)	0.5	−0.07 (−0.15, 0.00)	0.06
	Adjusted	−0.02 (−0.08, 0.04)	0.5	−0.07 (−0.15, 0.01)	0.09
Stack zone	Baseline model	0.01 (−0.05, 0.07)	0.8	−0.002 (−0.08, 0.07)	1.0
	Adjusted	0.01 (−0.04, 0.07)	0.6	0.004 (−0.07, 0.08)	0.9
***FEV1-post bronchodilator (L)*** a b					
Reduced exp zone	Baseline model	−0.03 (−0.12, 0.05)	0.5	−0.05 (−0.15, 0.05)	0.4
	Adjusted	−0.03 (−0.12, 0.06)	0.5	−0.05 (−0.15, 0.06)	0.4
Increased exp zone	Baseline model	0.02 (−0.07, 0.10)	0.7	−0.06 (−0.17, 0.04)	0.2
	Adjusted	0.01 (−0.07, 0.10)	0.8	−0.06 (−0.17, 0.04)	0.2
Stack zone	Baseline model	−0.08 (−0.17, 0.00)	0.06	−0.06 (−0.16, 0.05)	0.3
	Adjusted	−0.08 (−0.16, 0.01)	0.07	−0.05 (−0.16, 0.05)	0.3
***FEV1/FVC-pre bronchodilator*** b c					
Reduced exp zone	Baseline model	0.01 (−0.01, 0.03)	0.2	0.006 (−0.01, 0.03)	0.6
	Adjusted	0.01 (−0.01, 0.03)	0.2	0.005 (−0.01, 0.02)	0.6
Increased exp zone	Baseline model	0.002 (−0.02, 0.02)	0.8	−0.002 (−0.02, 0.02)	0.8
	Adjusted	0.002 (−0.02, 0.02)	0.8	−0.003 (−0.02, 0.02)	0.8
Stack zone	Baseline model	0.004 (−0.01, 0.02)	0.7	0.004 (−0.01, 0.02)	0.7
	Adjusted	0.004 (−0.01, 0.02)	0.7	0.005 (−0.01, 0.02)	0.6
***FEV1/FVC-post bronchodilator*** b c					
Reduced exp zone	Baseline model	0.009 (−0.00, 0.02)	0.2	0.003 (−0.01, 0.02)	0.6
	Adjusted	0.009 (−0.00, 0.02)	0.2	0.003 (−0.01, 0.02)	0.6
Increased exp zone	Baseline model	0.00 (−0.01, 0.01)	1.0	−0.009 (−0.02, 0.00)	0.2
	Adjusted	−0.001 (−0.01, 0.01)	0.9	−0.009 (−0.02, 0.00)	0.1
Stack zone	Baseline model	0.005 (−0.01, 0.02)	0.5	0.009 (−0.00, 0.02)	0.2
	Adjusted	0.005 (−0.01, 0.02)	0.5	0.009 (−0.00, 0.02)	0.1
***FVC-pre bronchodilator (L)*** a b					
Reduced exp zone	Baseline model	−0.04 (−0.12, 0.05)	0.4	−0.02 (−0.12, 0.08)	0.7
	Adjusted	−0.04 (−0.12, 0.05)	0.4	−0.02 (−0.12, 0.08)	0.8
Increased exp zone	Baseline model	−0.03 (−0.11, 0.05)	0.5	−0.09 (−0.19, 0.01)	0.09
	Adjusted	−0.03 (−0.11, 0.05)	0.5	−0.08 (−0.18, 0.02)	0.1
Stack zone	Baseline model	0.001 (−0.08, 0.08)	1.0	−0.02 (−0.11, 0.08)	0.8
	Adjusted	0.004 (−0.08, 0.08)	0.9	−0.01 (−0.11, 0.09)	0.8
***FVC-post bronchodilator (L)*** a b					
Reduced exp zone	Baseline model	−0.05 (−0.15, 0.05)	0.4	−0.05 (−0.16, 0.07)	0.4
	Adjusted	−0.04 (−0.14, 0.06)	0.4	−0.05 (−0.16, 0.07)	0.4
Increased exp zone	Baseline model	0.01 (−0.09, 0.11)	0.8	−0.05 (−0.16, 0.06)	0.4
	Adjusted	0.01 (−0.09, 0.11)	0.8	−0.05 (−0.16, 0.07)	0.4
Stack zone	Baseline model	−0.11 (−0.21, −0.01)	0.03	−0.09 (−0.20, 0.02)	0.1
	Adjusted	−0.10 (−0.20, −0.01)	0.04	−0.09 (−0.20, 0.02)	0.1
***Peak flow (L/min) morning*** a b d					
Reduced exp zone	Baseline model	−4.76 (−13.63, 4.11)	0.3	−5.74 (−16.90, 5.43)	0.3
	Adjusted	−5.20 (−14.40, 4.01)	0.3	−6.62 (−18.03, 4.79)	0.3
Increased exp zone	Baseline model	−2.56 (−11.85, 6.71)	0.6	−5.93 (−17.52, 5.65)	0.3
	Adjusted	−2.52 (−12.00, 6.95)	0.6	−4.56 (−16.46, 7.34)	0.5
Stack zone	Baseline model	−9.69 (−18.66, −0.72)	0.03	4.31 (−6.83, 15.45)	0.5
	Adjusted	−9.77 (−18.94, −0.60)	0.04	4.64 (−6.70, 15.97)	0.4
***Peak flow (L/min) evening*** a b d					
Reduced exp zone	Baseline model	−5.32 (−14.32, 3.67)	0.3	−6.93 (−18.39, 4.53)	0.2
	Adjusted	−5.29 (−14.61, 4.02)	0.3	−7.65 (−19.35, 4.06)	0.2
Increased exp zone	Baseline model	−0.83 (−10.24, 8.58)	0.9	−3.71 (−15.59, 8.18)	0.5
	Adjusted	−0.74 (−10.34, 8.85)	0.9	−1.82 (−14.02, 10.39)	0.8
Stack zone	Baseline model	−8.16 (−17.25, 0.94)	0.08	2.82 (−8.62, 14.25)	0.6
	Adjusted	−7.90 (−17.19, 1.38)	0.1	3.24 (−8.38, 14.86)	0.6
***Exhaled nitric oxide (eNO)*** b e		**Ratio (95% CI)**		**Ratio (95% CI)**	
Reduced exp zone	Baseline model	0.94 (0.75, 1.19)	0.6	0.89 (0.71, 1.12)	0.3
	Adjusted	0.93 (0.74, 1.17)	0.5	0.92 (0.73, 1.14)	0.4
Increased exp zone	Baseline model	1.05 (0.83, 1.32)	0.7	1.08 (0.86, 1.35)	0.5
	Adjusted	1.00 (0.79, 1.26)	1.0	1.09 (0.87, 1.36)	0.4
Stack zone	Baseline model	0.90 (0.72, 1.13)	0.4	0.90 (0.73, 1.13)	0.4
	Adjusted	0.91 (0.73, 1.14)	0.4	0.93 (0.75, 1.15)	0.5

a Baseline models adjusted for: age; age^2^; height; gender; gender*age; gender*age^2^; gender*height.

b All adjusted models additionally adjusted for: diagnosed asthma at baseline; smoking; gas cooker or oven; unflued gas heating; ETS; education and employment status.

c Baseline models adjusted for: age, gender, gender*age.

d PEF recorded three times twice daily at morning and evening.

e Adjusted for age and gender.

### Questionnaire

Questionnaires collected information on respiratory symptoms and diagnoses (wheeze, cough and asthma), eye, nose and throat symptoms, and use of medications, and on potential confounders and effect modifiers including smoking, environmental tobacco smoke (ETS) exposure, and use of gas heating and gas cooking in the home. The respiratory health questions were adapted from the ISAAC [7] and Belmont studies [8], with questions about eye, nose and throat symptoms from the M5East study [2]. We asked about symptoms experienced within the preceding three months (in contrast to 12 months for ISAAC) so that the recall period for 2007 did not extend into the pre-tunnel period. Questions on mouth symptoms in adults, which we did not expect to be associated with air pollution exposure, were used to assess reporting bias. We used questionnaire responses to construct composite variables for three primary outcomes: lower respiratory tract symptoms (LRS), severe LRS (SLRS), and upper respiratory tract symptoms (URS) (see Supporting information, Table S1).

**Table 6:** Odds ratios for symptoms reported in the diary panel sub-cohort in 2007 and in 2008 relative to 2006, adjusted for change in the control zone, (n = 380).

Symptoms measured by diary		2007 vs 2006		2008 vs 2006	
		Odds Ratio^a^	P	Odds Ratio^a^	P
		(95% CI)		(95% CI)	
***Day symptoms***					
Reduced exp zone	Baseline model	0.7 (0.2, 1.7)	0.4	0.6 (0.2, 1.6)	0.3
	Adjusted^b^	0.6 (0.2, 1.6)	0.3	0.5 (0.2, 1.4)	0.2
Increased exp zone	Baseline model	0.4 (0.1, 1.1)	0.06	0.4 (0.1, 1.3)	0.1
	Adjusted	0.3 (0.1, 0.9)	0.04	0.4 (0.1, 1.1)	0.09
Stack zone	Baseline model	1.1 (0.4, 2.9)	0.9	0.5 (0.2, 1.4)	0.2
	Adjusted	1.1 (0.4, 2.8)	0.9	0.4 (0.2, 1.3)	0.1
***Night symptoms***					
Reduced exp zone	Baseline model	1.0 (0.4, 2.7)	1.0	0.6 (0.2, 1.6)	0.3
	Adjusted	1.0 (0.4, 2.8)	1.0	0.5 (0.2, 1.4)	0.2
Increased exp zone	Baseline model	0.4 (0.2, 1.2)	0.1	0.5 (0.2, 1.4)	0.2
	Adjusted	0.5 (0.2, 1.3)	0.1	0.4 (0.1, 1.2)	0.1
Stack zone	Baseline model	1.4 (0.5, 3.8)	0.5	1.1 (0.4, 3.0)	0.9
	Adjusted	1.5 (0.5, 4.0)	0.5	1.0 (0.4, 2.8)	1.0
***Symptom days***					
Reduced exp zone	Baseline model	1.2 (0.4, 3.6)	0.7	0.6 (0.2, 2.0)	0.4
	Adjusted	1.1 (0.4, 3.4)	0.8	0.6 (0.2, 1.9)	0.4
Increased exp zone	Baseline model	0.5 (0.1, 1.4)	0.2	0.8 (0.3, 2.6)	0.7
	Adjusted	0.4 (0.1, 1.2)	0.1	0.6 (0.2, 2.0)	0.4
Stack zone	Baseline model	2.4 (0.8, 7.1)	0.1	1.8 (0.6, 5.6)	0.3
	Adjusted	2.5 (0.8, 7.5)	0.1	1.6 (0.5, 4.9)	0.4
***Upper respiratory symptoms***					
Reduced exp zone	Baseline model	0.6 (0.2, 1.6)	0.3	0.5 (0.2, 1.4)	0.2
	Adjusted	0.5 (0.2, 1.5)	0.2	0.4 (0.1, 1.2)	0.1
Increased exp zone	Baseline model	0.2 (0.07, 0.6)	0.005	0.1 (0.04, 0.4)	0.001
	Adjusted	0.2 (0.07, 0.6)	0.005	0.1 (0.04, 0.4)	<0.001
Stack zone	Baseline model	0.8 (0.3, 2.3)	0.7	0.3 (0.1, 0.9)	0.03
	Adjusted	0.8 (0.3, 2.2)	0.6	0.3 (0.1, 0.8)	0.02
***Wheeze***					
Reduced exp zone	Baseline model	1.2 (0.3, 4.0)	0.8	0.9 (0.3, 3.5)	0.9
	Adjusted	1.4 (0.4, 4.7)	0.6	1.1 (0.3, 4.0)	0.9
Increased exp zone	Baseline model	0.5 (0.1, 2.0)	0.3	1.2 (0.3, 4.6)	0.8
	Adjusted	0.5 (0.2, 1.9)	0.3	1.1 (0.3, 4.2)	0.9
Stack zone	Baseline model	2.2 (0.6, 7.5)	0.2	2.9 (0.8, 10.5)	0.1
	Adjusted	2.6 (0.8, 8.6)	0.1	3.3 (0.9, 11.6)	0.06
***Cough***					
Reduced exp zone	Baseline model	0.9 (0.3, 2.3)	0.8	0.4 (0.1, 1.1)	0.07
	Adjusted	0.8 (0.3, 2.2)	0.7	0.3 (0.1, 0.9)	0.03
Increased exp zone	Baseline model	0.4 (0.1, 1.1)	0.08	0.3 (0.1, 0.9)	0.03
	Adjusted	0.4 (0.1, 1.1)	0.06	0.2 (0.1, 0.7)	0.01
Stack zone	Baseline model	1.5 (0.5, 4.1)	0.4	0.6 (0.2, 1.8)	0.4
	Adjusted	1.5 (0.5, 4.1)	0.4	0.5 (0.2, 1.5)	0.2

a OR-odds of higher values on the ordinal scale (symptoms experienced: 0 days; 1–10% of days;10–20% of days; and 21–100% of days) compared with lower values, assuming proportional odds.

b Adjusted for: age; gender; diagnosis of asthma at baseline; smoker; ETS; gas stove or oven; unflued gas heating; education and employment status.

### Diary Panel Sub-cohort

In panel sub-cohort participants (n = 380) we performed allergen skin prick tests to define atopic status in 2007 and measured spirometric lung function (forced expiratory volume in one second (FEV_1_) and forced vital capacity (FVC)) and exhaled nitric oxide (eNO) once each year (2006–2008) at a home visit. We calculated the ratio of FEV_1_ as a proportion of FVC (FEV_1_/FVC) as a measure of airflow obstruction. We also asked participants to record symptoms, medication use and peak expiratory flows (PEF) twice daily for nine weeks each year at the same time of year (see Supporting information, Appendix S2).

**Table 7:** Mean pollutant concentrations by year and difference in pollutant concentration from post-tunnel year compared to pre-tunnel year.

Site & pollutant	Mean pollutant levels (SD)		Difference in pollutant levels^a^	
	Pre-tunnel year^a^	First post-tunnel year^b^	Unadjusted estimate (95% CI)	Adjusted estimate (95% CI)^c^
***Elevated site near eastern ventilation stack***				
NO_2_ ppb	16.7 (7.0)	15.7 (5.3)	−2.55 (−4.43, −0.66)	−1.00 (−2.21, 0.22)
NOx ppb	30.7 (17.8)	27.1 (14.1)	−6.33 (−10.97, −1.70)	−2.10 (−3.84, −0.37)
PM_10_ *µ*g/m^3^	20.2 (8.1)	16.1 (6.8)	−4.02 (−6.24, −1.80)	−1.74 (−3.04, −0.43)
PM_2.5_ *µ*g/m^3^	6.4 (3.6)	5.3 (3.2)	−1.13 (−2.38, 0.12)	−0.26 (−0.65, 0.12)
***Elevated site near western ventilation stack***				
NO_2_ ppb	13.4 (5.6)	12.1 (4.5)	−1.96 (−3.08, −0.84)	−1.07 (−1.68, −0.45)
NOx ppb	26.1 (19.9)	20.6 (14.6)	−6.05 (−10.47, −1.62)	−2.79 (−4.46, −1.11)
PM_10_ *µ*g/m^3^	18.7 (7.4)	16.2 (7.0)	−2.39 (−4.22, −0.56)	−0.46 (−1.94, 1.03)
PM_2.5_ *µ*g/m^3^	6.2 (3.5)	5.3 (3.3)	−1.02 (−1.98, −0.06)	−0.19 (−0.65, 0.27)

a Difference between pre-tunnel and first post-tunnel years, estimated by autoregressive analysis.

b Pre-tunnel is 25 March 06–24 March 07 and first post-tunnel year is 25 March 2007–24 March 2008.

Data for only one post-tunnel year available.

c Adjusted for change in air quality at the three Sydney regional sites (averaged).

### Air Quality Monitoring

Two elevated air quality monitoring stations located near the ventilation stacks measured oxides of nitrogen (NOx), nitric oxide (NO), NO_2_, particulate matter less than 2.5 *µ*m and less than 10 *µ*m in diameter respectively (PM_2.5_ and PM_10_) and carbon monoxide (CO). Monitoring methods were identical to those previously described for fixed site monitoring in the study area [9].

### Statistical Analysis

We compared the study respondents’ characteristics to the local population using Australian Census 2006 data [10].

Study hypotheses were tested using a regression model. Main study factors were study year, exposure zone and their interaction. We tested the hypotheses by fitting a contrast statement to this interaction term. This approach modelled changes in symptoms and lung function between pre-tunnel year (2006) and each post-tunnel year (2007 and 2008) by zone, adjusted for the change in the control zone. Coefficients for contrasts were reported as mean differences for the continuous variables, or odds ratios for binary or multinomial variables.

To adjust for autocorrelation we fitted random intercepts for subjects and households. An additional random intercept for study year was fitted for PEF data, due to repeated measures each year. Some models with both subject and household random effects did not converge, and we re-fitted the model with random intercepts for subject only.

We fitted both baseline models and models adjusted for covariates. All models, including baseline models, included adjustment for changes in the control zone. For adult questionnaire outcomes we fitted a model adjusting additionally for mouth symptoms to assess potential reporting bias. Covariates incorporated in adjusted models were specified *a priori* and included: asthma diagnosis at baseline; smoking status; ETS at home; use of gas cooking (stove or oven); use of unflued gas heaters; education; and employment status. Covariates were retained in these models if they contributed significantly (P<0.05) to the model.

Baseline models for FEV_1_, FVC and PEF were adjusted for height, age, age^2^, gender and their interaction, and models for FEV_1_/FVC were adjusted for age and gender and their interaction, based on models used in predictive equations [11]. Baseline models for eNO were adjusted for age and gender. The same covariates as incorporated for the questionnaire analysis were used and all covariates were retained in these models.

Continuous variables were tested using linear regression. The distribution of eNO was skewed so its values were log-transformed. Other continuous variables were analysed without transformation. Questionnaire symptoms were analysed as binary outcomes using logistic regression. For the individual daily panel symptom data, we attempted to fit a general linear mixed model incorporating random effects for subject and day with a logit link. As these models did not converge, we used summarised data for each subject, converting daily symptom data to the proportion of days each year the subject reported symptoms, classified on an ordinal scale as: no days; 1–10%; 11–20%; and 21–100% of days. These data were analysed using ordinal regression with a cumulative logit link and a multinomial error distribution. The same covariates as specified previously were incorporated and retained in the diary symptom models.

Linear regression and multinomial regression models were fitted in Proc MIXED and Proc GLIMMIX, respectively using SAS 9.2. The logistic regression with two random effects used for the questionnaire analysis was fitted in MLwiN using the Markov Chain Monte Carlo (MCMC) estimation method, as these models did not converge when using numerical methods such as adaptive quadrature.

We used an autoregressive model to test whether there was a difference in air quality at the elevated monitoring sites between 2006 and later years, adjusting for changes in regional air quality measured at three Sydney sites by the NSW Office of Environment and Heritage (OEH). Analysis was conducted in SAS 9.2 using the AUTOREG procedure.

## Results

We identified 5,614 households in all study zones in 2006 (Figure 2). Overall household participation rate at baseline was 33%, varying from 25% in the stack zone to 38% in the control zone (see Supporting information, Table S2). 20% of households refused to participate, 6% were ineligible and no contact could be made with 42% of households. Survey respondents had a similar distribution of demographic characteristics to the general population in the study area [10].

Of 2,978 cohort members who completed the 2006 questionnaire, 2,191 (74%) provided data in 2007 and 1,713 (58%) in 2008 (Figure 2). Loss to follow up was lower in the stack zone than the control zone (odds ratio (OR) 0.73, 95% CI 0.58–0.92 for 2007 and OR 0.82, 95% CI 0.67–1.01 for 2008) and higher in the reduced exposure zone than the control zone in 2008 (OR 1.28, 95% CI 1.04–1.57).

### Baseline Data and Description of Study Group Over Time

Proportions of adults and females in the entire cohort did not vary substantially between zones or over the study period (Table 1). The prevalence of reporting wheeze, cough, diagnosed and current asthma, and use of asthma medication was similar across all zones at baseline (Table 1). The reduced exposure zone and the stack zone had more university-educated people and people in paid work, compared with the increased exposure zone and control zone. The proportion of participants using unflued gas heaters and gas cooktops/ovens increased in all zones over the study period. The prevalence of diagnosed asthma, cough, LRS, URS, and mouth symptoms increased in 2007 and 2008 in all zones.

Supporting information, Table S3, presents baseline data, reported in 2006, for the cohort that participated in each year of the study. Remaining members of the cohort in 2007 and in 2008 were similar in most baseline characteristics to the initial cohort, suggesting loss-to-follow-up is unlikely to have biased comparisons between years.

Of subjects asked to participate in the panel sub-cohort, 40% refused, 4% intended moving residence, and almost 6% were ineligible due to language or age constraints (Figure 2). The sub-cohort included more children than the entire cohort (Table 2), and the proportion of subjects with atopy was relatively high across all zones (50–58% in 2007). Spirometric measurements were similar across all zones in all years, although values for FEV_1_ and FVC were slightly higher in subjects residing within the stack zone (Table 2). Participants in the stack zone and the reduced exposure zone had higher PEF readings in all years than participants from the other two zones. eNO readings were higher in 2007 than in 2006 and 2008, across all zones. Over the study period, participants in the stack zone reported increased prevalence of wheeze (at least once) and slightly more symptomatic days (>20% of days), whereas in other zones participants reported a decrease in these symptoms (Table 3). There was some year-to-year variation in the prevalence and frequency of diary symptoms, within each zone.

Supporting information, Table S4, presents baseline data, reported in 2006, for the panel sub-cohort that participated in each year. Remaining participants in 2007 and 2008 were similar in most baseline characteristics to the initial cohort except that, in all zones other than the increased exposure zone, those remaining had reported substantially more cough at baseline. Also, in the increased exposure zone and the stack zone, the remaining 2008 sub-cohort had reported slightly less “current asthma” and “asthma medication use” at baseline than the initial sub-cohort and those remaining in 2007. This suggests limited potential for selection bias due to loss-to-follow up in this sub-cohort.

### Questionnaire Symptom Analysis

Relative to 2006, the odds for URS in 2007 and in 2008 were significantly increased in the increased exposure zone and the stack zone, after adjusting for change in the control zone (Table 4). The odds for LRS and SLRS were significantly increased in 2008 and 2007 respectively, in the stack zone. The odds ratios for symptoms in the reduced exposure zone were generally at or below 1.0 in 2007 and 2008, although were not statistically significant. Adjusting for significant covariates did not change the estimates substantially (Table 4). Similarly, adjusting for mouth symptoms in the analysis of adult questionnaire responses ((model 2), made little difference to the effect estimates (model 1) (see Supporting information, Table S5).

### Diary Analysis

In the panel sub-cohort, mean post-bronchodilator FVC and morning PEF were significantly reduced among stack zone participants in 2007, compared with 2006, after adjusting for change in the control zone (Table 5). This decrease was present, although not significant, for evening PEF. There was much less evidence of a similar change between 2006 and 2008. Although there were decreases in FEV_1_ and FVC (pre-bronchodilator) in the increased exposure zone in 2008, they were not significant. There were no significant changes in lung function, airflow obstruction (FEV_1_/FVC) or eNO in the other exposure zones.

In contrast to the questionnaire findings for the entire cohort, odds for symptoms recorded by panel sub-cohort participants were generally lower in all three exposure zones for both post-tunnel years, after adjustment for the control zone (Table 6). No significant increases in symptom prevalence were observed in any zone. Odds ratios for symptom days and wheeze in the stack zone were substantially greater than 1 but the confidence intervals (CIs) around these estimates were very broad. Adjustment for covariates did not materially alter the findings.

After adjusting for changes in regional air quality, monitors at the elevated sites near the ventilation stacks recorded significant decreases in PM_10,_ NOx and NO_2_ in 2007 compared to 2006 (Table 7). Similarly, we previously showed using data from four additional ground level monitors that after adjusting for changes in regional air quality and meteorological conditions, PM_10,_ NOx and NO_2_ concentrations decreased in the eastern part of the study area after the tunnel opened [9].

## Discussion

We did not observe consistent evidence of an improvement in respiratory health among residents living near a major road which experienced a traffic volume reduction of 50% after opening of a road tunnel. Among residents living adjacent to tunnel feeder roads there was increased reporting of upper respiratory tract symptoms in the questionnaire survey but not in the sub-cohort who recorded symptoms in a diary. The most consistently observed change was in residents living within a 650 m radius of the eastern ventilation stack. After the tunnel opened these subjects reported more upper and lower respiratory tract symptoms and had evidence of lower spirometric function, particularly lower FVC. Significant adverse effects were seen only for the first year of tunnel use (2007) and not for the second year (2008), except for questionnaire reported lower respiratory symptoms.

The strengths of this study are the cohort intervention design, in which measurements were made in the same individuals before and after commencement of operation of the tunnel, the inclusion of both subjective and objective measures of respiratory health, adjustment for coincident trends in a control site and for time-varying confounders, and testing for reporting bias.

Apart from the stack zone findings, findings from the questionnaire survey of the entire cohort and the panel sub-cohort were not consistent. This is perplexing but points to limitations of questions and diary cards that rely on self-report. Using a summary annual measure of symptoms for each subject, rather than daily symptom scores, in the sub-cohort analysis resulted in reduced statistical power, possibly contributing to inconsistency in results, but does not explain odds ratios being in the opposite direction.

Recall or other reporting bias is a potential weakness of self-reporting, which we sought to address by asking adults about mouth symptoms. The reported prevalence of mouth symptoms increased in all zones and was greatest in the increased exposure zone and the stack zone, a pattern similar for wheeze and LRS, which might be suggestive of information bias. However, when we included mouth symptoms as a covariate in the analysis, the observed effects for other symptoms were not attenuated. Hence, we conclude that the observed associations are unlikely to be explained by reporting bias.

The previous health investigation on a different tunnel stack [2] did not find an association between modelled annual average NOx concentrations representing ground level exposures from stack emissions and self-reported eye, nose and throat symptoms assessed by telephone interview. That study did not assess other respiratory symptoms or include lung function measures, thus making it difficult to compare with findings from our study. A few traffic intervention studies have reported improvements in respiratory health outcomes including wheeze and peak flow [12], hospitalisations for asthma [13], emergency department presentations [14], eNO [15], and nose and eye symptoms [16], after redirection or closure of roads or implementation of traffic restrictions during major events. These health benefits were demonstrated despite somewhat modest reductions in air pollutant concentrations, aside from major reductions seen during the Beijing Olympic Games. However, other studies have not found an effect [17]. For this local traffic intervention we did not see substantial benefits in health outcomes along the bypassed main road.

The most likely explanation for the lack of change in respiratory outcomes where this was expected is that the traffic intervention did not result in substantial changes in TRAP for most subjects. Although NO_2_ concentrations decreased adjacent to the bypassed main road, these changes were smaller than predicted, probably due to lower than predicted traffic volume through the tunnel [9]. Furthermore, changes in NO_2_ decreased markedly with increasing distance from the affected roads [18], which is consistent with previous evidence of a rapid decay in NO_2_ concentrations with distance from major roads [19], [20].

The findings of this study suggest an adverse health effect associated with living in the stack zone. This is based on lower and upper respiratory tract symptoms and wheeze recorded by questionnaire in the entire cohort, lung function analysis of panel participants, and (albeit not significantly) diary symptom analysis. These findings related primarily, but not exclusively to 2007, the first year of tunnel operation. While the confidence intervals for the diary symptom scores were wide, the outcomes are consistent with other effects reported for the stack zone. These findings are unexpected, given that neither the modelled stack emissions nor measured NO_2_, PM_10_ or PM_2.5_ at the elevated monitors, showed any evidence of increased concentration after the tunnel commenced operation.

Bias of some kind as an explanation for positive associations cannot be ruled out in observational studies, although, as previously discussed, we believe reporting bias is an unlikely explanation. While bias due to differential loss to follow-up is possible, there was no evidence that the remaining cohort had a higher prevalence of diagnosed asthma, current asthma or wheeze at baseline compared to the complete cohort or that smoking, education or work status changed appreciably (see Supporting information, Tables S3 and S4). This suggests a lack of any major selective factors underlying loss to follow-up. Another possibility is that the findings are due to unmeasured confounding factors.

We considered whether the adverse effects in the stack zone could be due to exposure to tunnel portal emissions due to its location near the tunnel’s eastern portal, an area with potential to experience an increase in pollutant concentrations [4]. However, the LCT was designed not to allow for portal emissions. Furthermore, the residents in the ‘increased exposure zone’, also located near the tunnel portal, did not show the same pattern of adverse health effects. The observed effects in the stack zone might have been due to TRAP associated with a highway which traverses the zone, however, highway traffic decreased after 2006 [9].

Another possible explanation for the stack zone findings is an alternative pollutant source located in the industrial area near the zone. While the local government authority indicated that land zoning did not change in the industrial estate during the study timeframe [21], it does not keep records of processes undertaken on individual properties. Thus we cannot rule out this possibility but also have no direct evidence for it.

The adverse findings in the stack zone are also not attributable to any measured pollutants emitted from the eastern ventilation stack, as discussed above. In addition, previous analysis of air quality data showed a decrease in NO_2_, PM_10_ or PM_2.5_ in the eastern section of the study area, over the study period [9]. Furthermore, the choice of a 650 m buffer for the stack zone was based on sample size considerations, rather than on any modelled or measured pollutant profiles.

It is possible, though, that the adverse findings are due to pollutants or size fractions other than those measured. Measurements of PM_10_ probably do not adequately reflect the particulate content of emissions in tunnels [4] and some studies show that PM_10_ and PM_2.5_ do not represent TRAP as well as other pollutants such as NOx, NO_2_, black carbon or elemental carbon [22], [23]. Smaller size PM fractions that is, PM_1_ or PM_0.1_ (ultrafine particles (UFP)) are of lower mass, are present in far greater numbers, and, due to their oxidative potential, larger surface areas and ability to attach to other compounds, are considered to pose greater toxicity risks than PM_10_ [24]–[26]. Furthermore, elevated levels of UFP have been observed inside tunnels [27].

The finding of adverse respiratory health effects among residents living in the eastern stack zone is unexpected and difficult to explain, and as pollutant concentrations decreased in the immediate area, we are unable to attribute the effects to the ventilation stack. However, the findings imply the need to investigate the emission profile of the stack and other pollutant sources in the area that might explain the findings. The lack of improvement in respiratory health among people living along the bypassed main road is disappointing but probably reflects the short distance from the bypassed road within which exposure reduction might result in measurable health improvement.

## Supporting Information

Table S1 **Composite variables for LRS, SLRS and URS.**
(DOC)

Table S2 **Response rates for household participation and participant description at recruitment (2006), and comparison with general population (2006 Census).**
(DOC)

Table S3 **Baseline (2006) characteristics of the cohort members who participated, by zone and study year.**
(DOC)

Table S4 **Baseline (2006) characteristics of the diary panel sub-cohort who participated, by zone and year (n = 380).**
(DOC)

Table S5 **Odds ratios for symptoms reported by questionnaire in 2007 and in 2008, compared to 2006, without (model 1) and with (model 2) adjustment for potential reporting bias (adult subjects only).**
(DOC)

Appendix S1 **Definition and choice of zones.**
(DOC)

Appendix S2 **Measurement of lung function, eNO, atopy and sample size calculations.**
(DOC)
