# Supplementary material for: Respiratory Health before and after the Opening of a Road Traffic Tunnel: A Planned Evaluation
Source: PLoS One. 2012 Nov 29;7(11):e48921. doi: 10.1371/journal.pone.0048921 (PMC3510202; doi:10.1371/journal.pone.0048921)
Supplement: Table S1 — Composite variables for LRS, SLRS and URS. (DOC) [file pone.0048921.s001.doc]

**Table S1. Composite variables for LRS, SLRS and URS**

| **Composite variables^b^** | **Questionnaire variables^a^** | |
| --- | --- | --- |
|  | **Adult questions** | **Children’s questions** |
| **Lower respiratory symptoms (LRS)** | Wheeze | Wheeze |
|  | Asthma/wheeze limited activities | Asthma/wheeze limited activities |
|  | Medication for asthma | Medication for asthma |
|  | Cough + longest episode of cough ≥1 week | Cough + longest episode of cough ≥1 week |
|  | Cough + frequency of cough at least once/mth | Cough + frequency of cough at least once/mth |
|  | Night cough without URTI | Night cough without URTI |
|  | Night cough lasting>2 wks | Night cough lasting>2 wks |
|  | Chest tightness at anytime at least once/mth | Missed school |
|  | Chest tightness upon waking at least once/mth |  |
|  | Visited doctor or emergency department or admitted to hospital for asthma episode | Visited doctor or emergency department or admitted to hospital for asthma episode |
|  | Attacks of wheezing: >3 | Attacks of wheezing: >3 |
| **Severe lower respiratory symptoms (SLRS)** | Sleep disturbed due to wheezing: 1 or more nights/wk | Sleep disturbed due to wheezing: 1 or more nights/wk |
|  |  | Wheeze severe enough to limit speech |
|  | Asthma/wheeze limited activities: >once/week | Asthma/wheeze limited activities: >once/week |
|  | Tightness in chest at any time: 2 times/wk or more | Missed school because of asthma/wheeze |
|  | Tightness in chest on waking: 2 times/wk or more |  |
|  | Visited doctor or emergency department or admitted to hospital for asthma episode | Visited doctor, emergency department, admitted to hospital for asthma episode |
|  | Use of short-acting bronchodilator at least daily | Use of short-acting bronchodilator at least daily |
|  | Taken any oral prednisone | Taken any oral prednisone |
| **Upper respiratory symptoms (URS)** | Nasal symptoms without cold or flu (often or all the time) | Rhinitis (without cold or flu) (ever) |
|  | Dry or sore throat without cold or flu (often or all the time) | Rhinitis (without cold or flu) in last 3 mths |

**^a^** Occurrence of symptoms within last 3 months

**^b^**  Composite variables (LRS, SLRS, URS): If yes to any questionnaire symptom then composite symptom= ‘yes’; else composite symptom=’no’
