# Supplementary material for: Respiratory Health before and after the Opening of a Road Traffic Tunnel: A Planned Evaluation
Source: PLoS One. 2012 Nov 29;7(11):e48921. doi: 10.1371/journal.pone.0048921 (PMC3510202; doi:10.1371/journal.pone.0048921)
Supplement: Table S2 — Response rates for household participation and participant description at recruitment (2006), and comparison with general population (2006 Census). (DOC) [file pone.0048921.s002.doc]

**Table S2 Response rates for household participation and participant description at recruitment (2006), and comparison with general population (2006 Census)**

|  |  |  | **Children (2-17 yrs) %** | | **Adults (18-75 yrs) %** | | **Gender Male (%)** | |
| --- | --- | --- | --- | --- | --- | --- | --- | --- |
| **Zone** | **Total estimated households (n)** | **Household participation (%)** | **Study population** | **Census population** | **Study population** | **Census population** | **Study population** | **Census population** |
| Reduced exposure zone | 1598 | 37 | 29 | 23 | 70 | 77 | 44 | 46 |
| Increased exposure zone**^a^** | 1275 | 31 | 27 | 22 | 73 | 78 | 46 | 47 |
| Eastern stack zone**^a^** | 1465 | 25 | 26 | 20 | 74 | 80 | 47 | 49 |
| Control zone | 1274 | 38 | 29 | 26 | 70 | 74 | 47 | 49 |
| Overall | 5612 | 33 |  |  |  |  |  |  |

**^a^** Participants in overlapping area of increased exposure zone and eastern stack zone (n=48) contributed data to both zones
