# Supplementary material for: Respiratory Health before and after the Opening of a Road Traffic Tunnel: A Planned Evaluation
Source: PLoS One. 2012 Nov 29;7(11):e48921. doi: 10.1371/journal.pone.0048921 (PMC3510202; doi:10.1371/journal.pone.0048921)
Supplement: Table S3 — Baseline (2006) characteristics of the cohort members who participated, by zone and study year. (DOC) [file pone.0048921.s003.doc]

**Table S3 Baseline (2006) characteristics of the cohort members who participated, by zone and study year**

|  | **Reduced exposure zone** | | | **Increased exposure zone** | | | **Eastern stack zone** | | | **Control zone** | | |
| --- | --- | --- | --- | --- | --- | --- | --- | --- | --- | --- | --- | --- |
|  | **2006** | **2007** | **2008** | **2006** | **2007** | **2008** | **2006** | **2007** | **2008** | **2006** | **2007** | **2008** |
|  |  |  |  |  |  |  |  |  |  |  |  |  |
| **N (%)** | 1024 | 756 (74) | 608 (59) | 589 | 422 (72) | 302 (51) | 645 | 500 (78) | 394 (61) | 768 | 537 (70) | 424 (55) |
| **%** |  |  |  |  |  |  |  |  |  |  |  |  |
| Adults (18-75 yrs) | 72 | 70 | 68 | 74 | 72 | 71 | 75 | 72 | 72 | 72 | 72 | 72 |
| Females | 56 | 57 | 58 | 53 | 54 | 58 | 54 | 53 | 54 | 53 | 54 | 54 |
| Diagnosed asthma | 18 | 19 | 19 | 18 | 19 | 19 | 16 | 18 | 19 | 15 | 17 | 16 |
| Current asthma | 11 | 11 | 12 | 12 | 12 | 12 | 9 | 10 | 10 | 9 | 9 | 10 |
| Wheeze (ever) | 28 | 30 | 30 | 22 | 23 | 24 | 23 | 26 | 28 | 19 | 21 | 20 |
| Wheeze (last 3 mths) | 15 | 15 | 16 | 14 | 14 | 14 | 11 | 12 | 13 | 10 | 11 | 12 |
| Asthma med’n (last 3 mths) | 11 | 12 | 13 | 11 | 12 | 11 | 10 | 11 | 11 | 9 | 9 | 10 |
| Inhaled corticosteroids (last 3m) | 6 | 7 | 8 | 4 | 4 | 5 | 4 | 5 | 5 | 5 | 5 | 6 |
| Cough (last 3 mths) | 40 | 40 | 39 | 33 | 33 | 33 | 36 | 37 | 38 | 34 | 36 | 36 |
| Lower resp. symptoms (LRS) | 39 | 38 | 39 | 35 | 36 | 36 | 34 | 36 | 37 | 33 | 34 | 35 |
| Severe LRS | 10 | 10 | 11 | 12 | 12 | 12 | 8 | 9 | 9 | 8 | 8 | 10 |
| Upper resp. symptoms | 17 | 18 | 17 | 10 | 10 | 10 | 12 | 13 | 12 | 12 | 14 | 14 |
| Mouth symptoms | 14 | 15 | 14 | 9 | 10 | 9 | 11 | 12 | 13 | 13 | 13 | 12 |
| Smoker **^a^** | 7 | 6 | 6 | 12 | 10 | 10 | 11 | 9 | 8 | 12 | 11 | 10 |
| ***Home environmental factors*** |  |  |  |  |  |  |  |  |  |  |  |  |
| Unflued gas heater | 25 | 26 | 27 | 12 | 15 | 16 | 18 | 20 | 23 | 20 | 19 | 20 |
| Gas cooktop or oven | 52 | 53 | 54 | 25 | 30 | 32 | 44 | 49 | 51 | 42 | 43 | 42 |
| ETS at home | 7 | 7 | 7 | 14 | 13 | 13 | 8 | 7 | 6 | 8 | 8 | 8 |
| ***Educational status*** |  |  |  |  |  |  |  |  |  |  |  |  |
| Tertiary educated | 61 | 64 | 62 | 46 | 45 | 45 | 62 | 64 | 64 | 44 | 44 | 43 |
| High school/diploma graduates**^b^** | 31 | 29 | 30 | 43 | 44 | 43 | 31 | 29 | 31 | 43 | 43 | 45 |
| Up to middle school**^c^** | 8 | 7 | 8 | 11 | 11 | 13 | 7 | 7 | 5 | 13 | 12 | 12 |
| ***Work status*** |  |  |  |  |  |  |  |  |  |  |  |  |
| Paid Work | 74 | 77 | 76 | 66 | 65 | 66 | 75 | 75 | 76 | 71 | 69 | 68 |

**^a^** Participants aged 2-17 yrs assumed to be non-smokers

**^b^**  Included: Diploma/TAFE, and participants who responded “Other”

**^c^** Included participants who refused to respond
