# Supplementary material for: Respiratory Health before and after the Opening of a Road Traffic Tunnel: A Planned Evaluation
Source: PLoS One. 2012 Nov 29;7(11):e48921. doi: 10.1371/journal.pone.0048921 (PMC3510202; doi:10.1371/journal.pone.0048921)
Supplement: Table S4 — Baseline (2006) characteristics of the diary panel sub-cohort who participated, by zone and year (n = 380). (DOC) [file pone.0048921.s004.doc]

**Table S4 Baseline (2006) characteristics^a^ of the diary panel sub-cohort who participated, by zone and year (n=380)**

|  | **Reduced exposure zone** | | | **Increased exposure zone^b^** | | | **Eastern stack zone^b^** | | | **Control zone** | | |
| --- | --- | --- | --- | --- | --- | --- | --- | --- | --- | --- | --- | --- |
|  | **2006** | **2007** | **2008** | **2006** | **2007** | **2008** | **2006** | **2007** | **2008** | **2006** | **2007** | **2008** |
| **N** | 93 | 85 | 79 | 98 | 77 | 70 | 97 | 85 | 74 | 98 | 71 | 61 |
| **%** |  | | | | | | | | | | | |
| Adults | 46 | 42 | 41 | 48 | 44 | 43 | 45 | 42 | 46 | 52 | 54 | 56 |
| Females | 54 | 52 | 53 | 51 | 52 | 47 | 56 | 54 | 54 | 45 | 46 | 44 |
| Diagnosed asthma | 16 | 14 | 18 | 23 | 26 | 23 | 16 | 16 | 15 | 13 | 14 | 15 |
| Current asthma | 9 | 8 | 9 | 14 | 14 | 11 | 7 | 7 | 4 | 9 | 10 | 11 |
| Wheeze (ever) | 24 | 24 | 24 | 21 | 21 | 20 | 22 | 20 | 19 | 17 | 20 | 20 |
| Wheeze (last 3 mths) | 12 | 11 | 10 | 11 | 9 | 9 | 6 | 5 | 4 | 11 | 13 | 13 |
| Asthma med’n (last 3 mths) | 11 | 11 | 11 | 13 | 14 | 10 | 7 | 7 | 4 | 9 | 10 | 11 |
| Inhaled corticosteroids (last 3m) | 9 | 8 | 9 | 4 | 4 | 0 | 2 | 2 | 1 | 5 | 6 | 7 |
| Cough (last 3 mths) | 19 | 36 | 39 | 19 | 21 | 17 | 18 | 42 | 46 | 15 | 30 | 30 |
| Lower respiratory symptoms (LRS)^c^ | 37 | 34 | 37 | 26 | 27 | 23 | 38 | 40 | 41 | 31 | 27 | 26 |
| Severe LRS^c^ | 6 | 7 | 6 | 12 | 10 | 9 | 5 | 4 | 0 | 9 | 8 | 10 |
| Upper resp. symptoms^c^ | 12 | 12 | 13 | 5 | 6 | 6 | 11 | 11 | 10 | 8 | 7 | 5 |
| Smoker ^d^ | 2 | 2 | 1 | 10 | 4 | 6 | 5 | 4 | 4 | 8 | 8 | 10 |
| ***Environmental factors*** |  |  |  |  |  |  |  |  |  |  |  |  |
| Unflued gas heater | 31 | 33 | 34 | 12 | 16 | 14 | 25 | 22 | 24 | 21 | 15 | 15 |
| Gas cooktop or oven | 57 | 58 | 61 | 26 | 29 | 33 | 63 | 62 | 62 | 43 | 34 | 34 |
| ETS at home | 6 | 7 | 6 | 15 | 8 | 10 | 2 | 0 | 1 | 4 | 4 | 5 |
| ***Educational status*** |  |  |  |  |  |  |  |  |  |  |  |  |
| Tertiary educated | 72 | 72 | 72 | 44 | 51 | 50 | 63 | 66 | 69 | 42 | 39 | 36 |
| High school/diploma graduates^e^ | 25 | 25 | 24 | 38 | 34 | 34 | 31 | 28 | 30 | 47 | 46 | 48 |
| Up to middle school^f^ | 3 | 4 | 4 | 18 | 16 | 16 | 6 | 6 | 1 | 11 | 14 | 16 |
| ***Work status*** |  |  |  |  |  |  |  |  |  |  |  |  |
| Paid Work | 80 | 80 | 81 | 60 | 62 | 59 | 82 | 86 | 84 | 74 | 70 | 70 |

**^a^** Measured by questionnaire in 2006

**^b^** Participants in overlapping area of increased exposure zone and eastern stack zone: diary cohort (2006=6; 2007=5; 2008=5) contributed data to both zones

^c^ Composite variables measured from questionnaire data

^d^ Participants aged 2-17 yrs assumed to be non-smokers

^e^ Included: Diploma/TAFE, and participants who responded “Other”

^f^ Included participants who refused to respond
