# Supplementary material for: Respiratory Health before and after the Opening of a Road Traffic Tunnel: A Planned Evaluation
Source: PLoS One. 2012 Nov 29;7(11):e48921. doi: 10.1371/journal.pone.0048921 (PMC3510202; doi:10.1371/journal.pone.0048921)
Supplement: Table S5 — Odds ratios for symptoms reported by questionnaire in 2007 and in 2008, compared to 2006, without (model 1) and with (model 2) adjustment for potential reporting bias (adult subjects only). (DOC) [file pone.0048921.s005.doc]

**Table S5 Odds ratios for symptoms reported by questionnaire in 2007 and in 2008, compared to 2006, without (model 1) and with (model 2) adjustment for potential reporting bias (adult subjects only)**

| **Outcomes measured by questionnaire** | | | **2007 vs 2006** | | **2008 vs 2006** | |
| --- | --- | --- | --- | --- | --- | --- |
|  | | | **Odds Ratio** | **P** | **Odds Ratio** | **P** |
|  | | | **(95% CI)** |  | **(95% CI)** |  |
| ***Upper respiratory symptoms*** | | | | | | |
| Reduced exposure zone | Model 1**^a^** | | 0.8 (0.4, 1.4) | 0.4 | 0.7 (0.4, 1.2) | 0.2 |
|  | Model 2**^b^** | | 0.8 (0.4, 1.4) | 0.4 | 0.6 (0.4, 1.2) | 0.1 |
| Increased exposure zone | Model 1 | | 1.3 (0.7, 2.6) | 0.4 | 1.6 (0.8, 3.3) | 0.2 |
|  | Model 2 | | 1.3 (0.6, 2.5) | 0.5 | 1.5 (0.7, 3.0) | 0.3 |
| Eastern stack zone | Model 1 | | 1.6 (0.8, 2.9) | 0.2 | 1.3 (0.7, 2.5) | 0.4 |
|  | Model 2 | | 1.5 (0.8, 2.8) | 0.2 | 1.2 (0.6, 2.3) | 0.6 |
| ***Lower respiratory symptoms*** | | | | | | |
| Reduced exposure zone | | Model 1 | 1.1 (0.7, 1.8) | 0.5 | 1.1 (0.7, 1.7) | 0.8 |
|  | | Model 2 | 1.1 (0.7, 1.8) | 0.6 | 1.0 (0.6, 1.7) | 1.0 |
| Increased exposure zone | | Model 1 | 1.1 (0.7, 1.9) | 0.6 | 1.1 (0.6, 1.8) | 0.9 |
|  | | Model 2 | 1.1 (0.7, 1.8) | 0.7 | 1.0 (0.6, 1.8) | 1.0 |
| Eastern stack zone | | Model 1 | 1.6 (1.0, 2.6) | 0.05 | 1.8 (1.05, 3.0) | 0.03 |
|  | | Model 2 | 1.5 (1.0, 2.4) | 0.08 | 1.7 (1.0, 2.9) | 0.049 |
| ***Severe lower respiratory symptoms*** | | | | | | |
| Reduced exposure zone | | Model 1 | 1.2 (0.5, 2.5) | 0.7 | 0.7 (0.3, 1.6) | 0.4 |
|  | | Model 2 | 1.2 (0.6, 2.6) | 0.7 | 0.7 (0.3, 1.6) | 0.4 |
| Increased exposure zone | | Model 1 | 1.0 (0.4, 2.2) | 0.9 | 0.8 (0.3, 1.8) | 0.6 |
|  | | Model 2 | 0.9 (0.4, 2.1) | 0.8 | 0.8 (0.3, 1.8) | 0.6 |
| Eastern stack zone | | Model 1 | 3.2 (1.4, 7.1) | 0.005 | 1.4 (0.6, 3.1) | 0.5 |
|  | | Model 2 | 3.1 (1.4, 6.9) | 0.006 | 1.3 (0.6, 2.9) | 0.5 |
| ***Wheeze in last 3 months*** | | | | | | |
| Reduced exposure zone | | Model 1 | 0.9 (0.5, 1.7) | 0.7 | 0.5 (0.2, 1.0) | 0.06 |
|  | | Model 2 | 0.9 (0.5, 1.8) | 0.8 | 0.5 (0.2, 1.1) | 0.07 |
| Increased exposure zone | | Model 1 | 0.9 (0.4, 1.8) | 0.7 | 1.1 (0.5, 2.5) | 0.8 |
|  | | Model 2 | 0.8 (0.4, 1.7) | 0.6 | 1.1 (0.5, 2.5) | 0.9 |
| Eastern stack zone | | Model 1 | 1.7 (0.9, 3.4) | 0.1 | 1.4 (0.6, 3.0) | 0.4 |
|  | | Model 2 | 1.7 (0.8, 3.3) | 0.2 | 1.4 (0.6, 3.0) | 0.5 |
| ***Cough in last 3 months*** | | | | | | |
| Reduced exposure zone | | Model 1 | 1.1 (0.7, 1.7) | 0.7 | 1.0 (0.6, 1.6) | 1.0 |
|  | | Model 2 | 1.1 (0.7, 1.6) | 0.7 | 1.0 (0.6, 1.6) | 0.9 |
| Increased exposure zone | | Model 1 | 1.1 (0.7, 1.7) | 0.8 | 0.8 (0.5, 1.4) | 0.5 |
|  | | Model 2 | 1.0 (0.6, 1.6) | 0.9 | 0.8 (0.5, 1.4) | 0.4 |
| Eastern stack zone | | Model 1 | 1.4 (0.9, 2.2) | 0.1 | 1.4 (0.9, 2.4) | 0.2 |
|  | | Model 2 | 1.4 (0.9, 2.1) | 0.2 | 1.4 (0.8, 2.3) | 0.2 |

**^a^** Model 1: Adjusted for doctor diagnosed asthma at baseline; smoking; gas cooker or oven; unflued gas heating; ETS in the home; education and employment status

**^b^** Model 2: Additionally adjusted for reporting of mouth symptoms to assess potential for reporting bias
