# Supplementary material for: Respiratory Health before and after the Opening of a Road Traffic Tunnel: A Planned Evaluation
Source: PLoS One. 2012 Nov 29;7(11):e48921. doi: 10.1371/journal.pone.0048921 (PMC3510202; doi:10.1371/journal.pone.0048921)
Supplement: Appendix S1 — Definition and choice of zones. (DOC) [file pone.0048921.s006.doc]

**Supporting information**

**Appendix SA. Definition and choice of zones**

The “predicted decreased exposure zone” and “predicted increased exposure zone” were defined using dispersion modelled data (Cal3qhc and Calmet/Calpuff models) available from the tunnel planning process . The modelling estimated small changes in pollutant concentrations arising from redistribution of traffic. For PM_10_, the change (pre-tunnel vs 2016 scenarios based on 100,000 vpd) ranged from 2% improvement to 2% deterioration. For NO_2_, the predicted change ranged from more than 10% deterioration up to 40% improvement. Given the much larger predicted changes for NO_2_ we used those modelled contours to define these two zones.

The eastern ventilation stack zone was chosen to address community concern about stack emissions, which existed despite the dispersion modelled results showing that under worst case conditions stack emissions would contribute little to background concentrations of NO_2_, PM_10_ and PM_2.5_. A zone with 650m radius around the eastern stack was chosen to ensure a sufficient sample size and because there were few homes within the same radius of the western stack. A small part of the “predicted increased exposure zone” at the eastern end of the study area and the eastern stack zone overlapped, hence some subjects were ascribed to both zones.

The control zone was in a nearby suburb, away from the influence of the tunnel and the bypassed main road. It was not expected to experience a change in TRAP over the study period.
