# Supplementary material for: Respiratory Health before and after the Opening of a Road Traffic Tunnel: A Planned Evaluation
Source: PLoS One. 2012 Nov 29;7(11):e48921. doi: 10.1371/journal.pone.0048921 (PMC3510202; doi:10.1371/journal.pone.0048921)
Supplement: Appendix S2 — Measurement of lung function, eNO, atopy and sample size calculations. (DOC) [file pone.0048921.s007.doc]

**Appendix SB**

**Measurement of lung function, eNO, atopy and sample size calculations**

***Spirometric lung function***

Spirometric lung function (forced expiratory volume in one second (FEV1) and forced vital capacity (FVC)) was measured using a hand held spirometer (QRS Diagnostic: Z-5000-2668) linked to Office Medic software (Version 4.5i). Spirometry was performed according to the American Thoracic Society/European Respiratory Society (ATS/ERS) criteria before, and 10 minutes after, the administration of bronchodilator (albuterol 200μg) via large volume spacer. Technically unsatisfactory curves were excluded from analysis. To improve reliability of measurements for the repeated measures study design we chose the higher of two reproducible measures within 150 ml as values for FEV_1_ and FVC. Where curves were not reproducible they were rechecked for quality, and when acceptable, the highest available measures for FEV_1_ and FVC were selected.

***Exhaled nitric oxide***

Exhaled nitric oxide (eNO) was collected as a marker of airway inflammation using an off-line technique according to ATS criteria and as previously described . Exhaled breath was collected into a 3L chemically inert bag through a rotameter incorporating a scrubber to ensure that ambient NO levels did not influence personal eNO concentrations. The NO concentration was measured using a chemiluminescence analyser (ThermoEnvironmental 42^o^C) within 24 hours of collection.

***Atopy-skin prick testing***

Skin prick testing was conducted during 2007 as previously described to test for atopy to the inhalant allergens: house dust mites (D. pteronyssinus, D. farinae), mould (Alternaria), cat pelt, dog, cockroach, ryegrass, and grass mix (Hollister-Stier). Wheal sizes were measured at 15 minutes as the average of the largest diameter and its perpendicular. Wheals larger than 3mm in diameter and larger than the negative control were considered to be positive. Subjects were classified as atopic if they had any positive skin prick test to an allergen.

***Height and weight***

Height and weight were measured at home visits, using a stadiometer and portable bathroom scales.

***Peak flow measurement***

Participants were asked to complete a twice daily peak flow and symptom diary for nine weeks each year, starting at the home visit. They used a Mini-Wright peak flow meter, after instruction on its use, to collect three peak expiratory flow (PEF) readings each morning and night. The highest value for each session was used for analysis, after excluding data for subjects with <14 days of full PEF data. Values more than four standard deviations above the mean were excluded.

Daily symptom recording included incidence and severity of cough, wheeze, dyspnea, runny or blocked nose, eye irritation, fever and/or sore throat, and use of reliever or preventer medication. Diaries were collected at the same time each study year, with the aim to follow-up participants within two weeks of their 2006 home visit date. If subjects were away from home, they were asked not to complete the diary for that period.

***Sample size***

*Questionnaire survey*

A sample size of 370 subjects per zone would provide 80% power (alpha=0.05) to detect a 5.5% change in the prevalence of wheeze between before and after tunnel opening. Allowing for a 25% loss to follow-up we aimed to recruit 500 subjects from each zone, totalling 2000 subjects.

*Panel study*

We estimated it would be feasible to recruit 90 subjects in each zone and anticipated that 60% of these (54) would be retained. We expected that each participant would have 63 repeated measures of PEF during each 9 week study period each year. Assuming the within-subject SD for PEF was 80 ml and autocorrelation in PEF measures was 0.6 we expected to have 80% power to detect a 36 ml difference in mean PEF between each exposure zone compared to the control zone, comparing pre-tunnel to post-tunnel years.
